# Outcomes following Pancreatoduodenectomy in Patients ≥80 Years: An Analysis of the Prospective Pancreas Registry of the German Society for General and Visceral Surgery (DGAV|Registry Pancreas)

**DOI:** 10.1159/vis/aeyag003

**Published:** 2026-10-10

**Authors:** Philipp Scherber, Sebastian Holländer, Gereon Gäbelein, Dorian Igna, Peter Jacob, Andreas Pascher, Michael Ghadimi, Christoph Reissfelder, Stefan Fichtner-Feigl, Jörg-Peter Ritz, Tobias Keck, Waldemar Uhl, Carsten Klinger, Heinz Buhr, Jörg C. Kalff, Jens Werner, Matthias Glanemann

**Affiliations:** a Department of General Surgery, Visceral-, Vascular- and Paediatric Surgery, Saarland University Medical Center, Homburg, Germany; b Department of General, Visceral and Transplant Surgery, University Hospital Münster, Münster, Germany; c Department of General, Visceral and Paediatric Surgery, University Medical Center Göttingen, Göttingen, Germany; d Department of Surgery, University Medical Center Mannheim, Medical Faculty Mannheim, Heidelberg University, Mannheim, Germany; e Department of General and Visceral Surgery, University of Freiburg Medical Center, Faculty of Medicine, Freiburg, Germany; f Department of General and Visceral Surgery, Helios Kliniken Schwerin, Schwerin, Germany; g Department of General Surgery, University Medical Center Schleswig Holstein, Campus Lübeck, Lübeck, Germany; h Department of General and Visceral Surgery, St. Josef- Hospital Bochum, Ruhr University Bochum, Bochum, Germany; i German Society for General and Visceral Surgery (DGAV), Haus der Bundespressekonferenz, Berlin, Germany; j Department for General, Visceral, Thoracic and Vascular Surgery, University Hospital of Bonn, Bonn, Germany; k Department of General, Visceral and Transplantation Surgery, Ludwig-Maximilians-University Hospital Munich, Munich, Germany

**Keywords:** Pancreatoduodenectomy, Pancreas registry, Octogenarians, DGAV, Mortality

## Abstract

**Introduction:**

With rising life expectancy, the number of elderly patients undergoing pancreatoduodenectomy is steadily increasing. This study aimed to assess peri- and postoperative outcomes in patients aged ≥ 80 years undergoing pancreatoduodenectomy, using data from the DGAV|registry pancreas.

**Methods:**

All pancreatoduodenectomies performed between September 2013 and April 2024 and recorded in the DGAV|registry pancreas were retrospectively analyzed. Patients were divided into two groups: <80 and ≥80 years. Demographic, perioperative, and postoperative data were compared. A multivariate analysis was conducted to identify independent risk factors for in-hospital mortality.

**Results:**

Out of 12,305 pancreatoduodenectomies, 1,566 (12.7%) were performed in patients aged ≥ 80 years. The proportion of very elderly patients more than doubled from 6.9% in 2014 to a peak of 17.3% in 2021. Malignant diseases, particularly pancreatic ductal adenocarcinoma, were more common in this group. While overall complication rates were slightly higher in the elderly (64.0% vs. 60.7%, *p* = 0.013), there was no significant difference in severe complications (Clavien-Dindo grades 3 and 4). However, 30-day mortality (7.2% vs. 3.6%) and in-hospital mortality (8.2% vs. 4.4%) were significantly higher in patients aged ≥ 80 years. Cardiopulmonary complications were also more frequent. Multivariate analysis identified age ≥ 80, male sex, ASA class 3 or 4, obesity, COPD, anticoagulant therapy, the need for intraoperative blood transfusions, and extended resections as independent predictors of in-hospital mortality.

**Conclusion:**

Chronological age ≥ 80 years is associated with increased postoperative morbidity and mortality after pancreatoduodenectomy. However, age alone should not be considered an exclusion criterion. Careful patient selection, taking biological age, comorbidities, and functional status into account, is essential. Integrating geriatric expertise and prehabilitation strategies may improve outcomes in this growing patient population.

## Introduction

Pancreatoduodenectomy is the standard surgical procedure for the treatment of both malignant and benign diseases around the pancreatic head, duodenum, and distal bile duct [1]. Due to improvements in perioperative and postoperative care, mortality rates have dropped to less than 5% in specialized centers in recent decades [2, 3]. However, reported morbidity is still very heterogeneous, ranging up to more than 40% [4–6].

Due to increasing life expectancy and the aging population in Western countries, an increasing number of elderly patients, particularly those ≥ 80 years, will present with diseases requiring pancreatoduodenectomy – for example, pancreatic ductal adenocarcinoma, which is both a typical disease of the elderly and the most common indication for pancreatoduodenectomy [7, 8].

In surgical literature, patients aged 80 years and older are commonly referred to as “very elderly” and represent a clinically distinct subgroup characterized by a disproportionately higher burden of comorbidities, frailty, and perioperative risk compared with younger patients [6, 9]. Given the rapidly increasing proportion of patients in this age category and their limited representation in previous studies, we deliberately focused on this subgroup to specifically assess peri- and postoperative outcomes following pancreatoduodenectomy [10].

The “Study, Documentation and Quality Center” (StuDoQ) was established by the German Society for General and Visceral Surgery (DGAV) in 2008 with the aim of transparent, evidence-based and risk-adapted recording and evaluation of treatment quality. On September 1, 2013, the database was expanded to include DGAV|registry pancreas. All participating centers for pancreatic surgery in Germany submit their patients’ treatment data to this registry, which enables both a general assessment of treatment quality and a comparison between the centers [11].

The aim of the present study was to verify the initial assumption of an increasing number of pancreatoduodenectomies, especially in very elderly patients aged ≥ 80 years, based on the DGAV|registry pancreas. In addition, peri- and postoperative outcomes were examined with regard to patient age (< or ≥ 80 years at the time of surgery) and potential risk factors for observed differences should be identified.

## Methods

The DGAV|registry pancreas of the German Society for General and Visceral Surgery was searched for this retrospective study. All pancreatoduodenectomies, including classical Whipple procedures and pylorus-preserving pancreatoduodenectomies (pp-Whipple), performed between September 2013 and April 2024 were included in the analysis – regardless of indication or the dignity of the underlying diseases. In accordance with the objectives of the study, the overall cohort was divided into patients younger than 80 years and very elderly patients aged 80 years and older at the time of surgery. The age threshold of 80 years was deliberately chosen to focus on a clinically distinct high-risk population that is consistently associated with increased comorbidity burden and perioperative vulnerability in major abdominal surgery. No further age stratification was performed, as the primary aim of the analysis was to assess whether very advanced chronological age per se represents an independent risk factor for adverse postoperative outcomes following pancreatoduodenectomy.

The patient characteristics collected included age, gender, body mass index, and the severity of comorbidities (classified according to the ASA scoring system of the American Society of Anesthesiologists). Information on relevant concomitant medications such as anticoagulants or immunosuppressants and the tumor marker CA 19-9 were recorded and considered as preoperative risk factors. In addition, intraoperative data on the type and extent of resection (classical or pylorus-preserving Whipple procedure, extended resections) were analyzed. Postoperative outcomes included the overall complication rate, the rate of severe postoperative complications (Clavien-Dindo grades 3 and 4), as well as specific surgical and nonsurgical complications. The postoperative length of stay in the intensive care unit and in the hospital, in-hospital mortality, and 30-day mortality were recorded. In DGAV|registry pancreas, death due to underlying disease, due to surgical or nonsurgical complications, or due to other/unknown causes were available as selectable variables to specify the reasons for the 30-day mortality. The study was submitted to the Ethics Committee of the Medical Association of Saarland (Ärztekammer des Saarlandes) and received approval (reference number: 158/25, date: 10 September 2025).

### Statistics

Data are presented as means or medians, standard deviations, and frequencies. Student’s *t*-test was used for normally distributed continuous variables; otherwise, the Mann-Whitney U test was applied.

Chi-square or Fisher’s exact tests were used for categorical variables. A two-sided *p* value ≤0.05 was considered statistically significant. Variables that showed a strong correlation with in-hospital mortality (*p* < 0.10) were subsequently tested in multivariate model. To address baseline heterogeneity between patients aged ≥ 80 years and younger patients, a propensity score-matched sensitivity analysis was performed. Patients were matched 1:1 using nearest-neighbor matching without replacement and a caliper of 0.2 standard deviations of the logit of the propensity score. The propensity score model included baseline demographic parameters and comorbidities. Covariate balance was assessed using standardized mean differences, with values <0.1 considered indicative of adequate balance. Outcomes were analyzed using conditional logistic regression with matched pairs as strata, and results are reported as odds ratios with 95% confidence intervals and *p* values. Statistical analysis was performed using SPSS software, version 29 (SPSS Inc., Chicago, IL).

## Results

Between September 2013 and April 2024, a total of 12,305 pancreatoduodenectomies were recorded in the DGAV|registry pancreas, of which 1,566 were performed in patients aged 80 years or older (group 2). As shown in Figure 1, the number of registered pancreatoduodenectomies increased steadily, reaching a peak of 1,587 surgeries in 2021. On average, around one in ten pancreatoduodenectomies was performed on a very elderly patient, with the proportion increasing from 6.9% in 2014 to 17.3% in 2021 (as shown in Fig. 1).

**Fig. 1.**
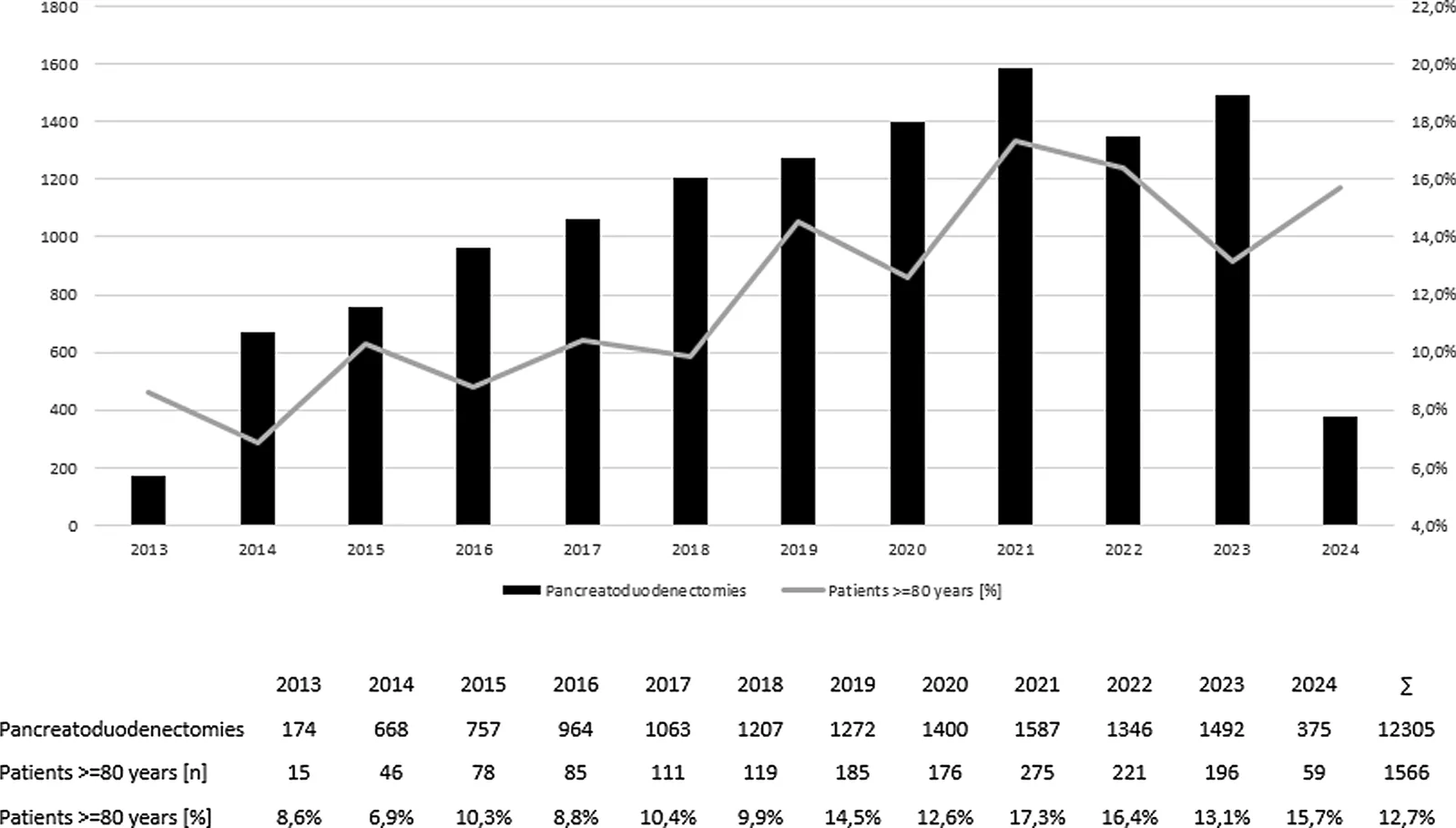
Pancreatoduodenectomies 2013–2024.

The median age of patients in the younger cohort <80 years (group 1) was 66 years, whereas in group 2, it was 82 years (Table 1). While the gender distribution in the group of very elderly patients was balanced, men predominated in the group of younger patients. Younger patients were significantly more likely to be overweight (48.8% vs. 42.8%, *p* < 0.001), whereas the prevalence of underweight was similar in both groups. Patients ≥ 80 years had significantly higher rates of concomitant cardiovascular diseases, e.g., heart failure, coronary artery disease, cerebrovascular events, and diabetes mellitus, which is reflected in the ASA classification: 69.2% of very elderly patients vs. 50.1% of younger patients were categorized in ASA class 3 or 4 (*p* < 0.001) (Table 1).

**Table 1.** Demographic parameters

​	Patients < 80 years	Patients ≥ 80 years	*p* value
*N*	10,739	1,566	​
Age (med [min/max])	66 (13/79) years	82 (80/93) years	**<0.001**
Men/women ratio	6,135/4,604 (57/43%)	777/789 (50/50%)	**<0.001**
BMI (med [min/max])	24.91 (11.40/59.50) kg/m^2^	24.49 (10.90/52.10) kg/m^2^	**<0.001**
BMI <18.5 kg/m^2^, *n* (%)	393 (3.7)	51 (3.3)	0.425
BMI >25 kg/m^2^, *n* (%)	5,239 (48.8)	61 (42.8)	**<0.001**
CA 19-9 (med [min/max])	44.00 (0.07/60,454) U/mL	75.00 (0.60/70,000) U/mL	**<0.001**
ASA 3/4, *n* (%)	5,377 (50.1)	1,081 (69.2)	**<0.001**
Comorbidities, *n* (%)			
Heart failure	1,074 (10.0)	313 (20.0)	**<0.001**
Coronary heart disease	1,268 (12.0)	367 (23.4)	**<0.001**
COPD	548 (5.1)	69 (4.4)	0.238
Cerebrovascular event	521 (4.9)	132 (8.4)	**<0.001**
End-stage renal disease	32 (0.3)	9 (0.6)	0.076
Peripheral arterial disease	234 (2.2)	36 (2.3)	0.762
Diabetes mellitus	2678 (24.9)	471 (30.1)	**<0.001**
Liver cirrhosis	254 (2.4)	27 (1.7)	0.347
Anticoagulant therapy	1,917 (17.99)	456 (29.7)	**<0.001**
Immunosuppressive therapy	114 (1.1)	15 (1.0)	0.707

The pylorus-preserving pancreatoduodenectomy (Traverso-Longmire) was the predominant surgical procedure in both groups (≈70%). With more than 80% in both groups, pancreatojejunostomy was the predominant type of pancreatic anastomosis (81.1% in group 1 vs. 84.7% in group 2, *p* = 0.002). Nearly all pancreatoduodenectomies (97% in both groups) were performed primarily as open surgery or converted to open surgery. The median operation time was slightly shorter in group 2 compared with group 1 (306 vs. 320 min, *p* < 0.001) (Table 2).

**Table 2.** Intra-, perioperative parameters

​	Patients < 80 years	Patients ≥ 80 years	*p* value
Surgical procedure (pp-Whipple/classical Whipple)	7,761/2978 (72.3/27.7%)	1,112/454 (71.0/29.0%)	0.299
Pancreatic anastomosis (pancreatojejuno-/pancreatogastrostomy)^a^	8,706/1,887 (81.1/17.6%)	1,326/225 (84.7/14.4%)	0.002
Surgical approach (open/minimally invasive)	10,453/286 (97.3/2.7%)	1,524/42 (97.3/2.7%)	0.501
Operation time (med [min/max])	320 (132/998)	306 (206/709)	**<0.001**
Intraoperative blood transfusion rate, *n* (%)	970 (9.0)	184 (11.7)	**<0.001**
Resection portal vein, *n* (%)	1,166 (10.9)	167 (10.7)	0.818
Resection truncus coeliacus^b^, *n* (%)	34 (0.3)	6 (0.4)	0.666
Extended resection, *n* (%)	893 (8.3)	111 (7.1)	0.097
Spleen	77 (0.7)	9 (0.6)	0.528
Kidney	39 (0.4)	2 (0.1)	0.131
Small intestine	359 (3.3)	55 (3.5)	0.729
Colon	211 (2.0)	23 (1.5)	0.179
Liver	287 (2.7)	30 (1.9)	0.077
Malignant disease, *n* (%)	7,678 (71.5)	1,314 (83.9)	**<0.001**
PDAC	5,206 (48.5)	911 (58.2)	**<0.001**
Chronic pancreatitis	1,123 (10.5)	26 (1.7)	**<0.001**

^a^ Missing values coded as “unknown” or “blind closure of the pancreatic stump.”

^b^ Resection of the “truncus coeliacus” refers to highly selected extended pancreatoduodenectomies in which resection of the truncus coeliacus was performed in conjunction with pancreatoduodenectomy, followed by reconstruction of the hepatic arterial inflow (e.g., hepatic artery reconstruction).

Histopathological analysis of the specimens showed that malignant diseases were significantly more frequently the indication for pancreatoduodenectomy in patients ≥80 years of age (83.9% vs. 71.5%, *p* < 0.001). Pancreatic ductal adenocarcinoma was the most common indication for pancreatoduodenectomy in both groups (48.5% in group 1 vs. 58.2% in group 2, *p* < 0.001). Chronic pancreatitis, in contrast, was the second most common indication among younger patients (10.5%) but was rare in patients ≥80 years (1.7%, *p* < 0.001) (as shown in Table 2; Fig. 2).

**Fig. 2.**
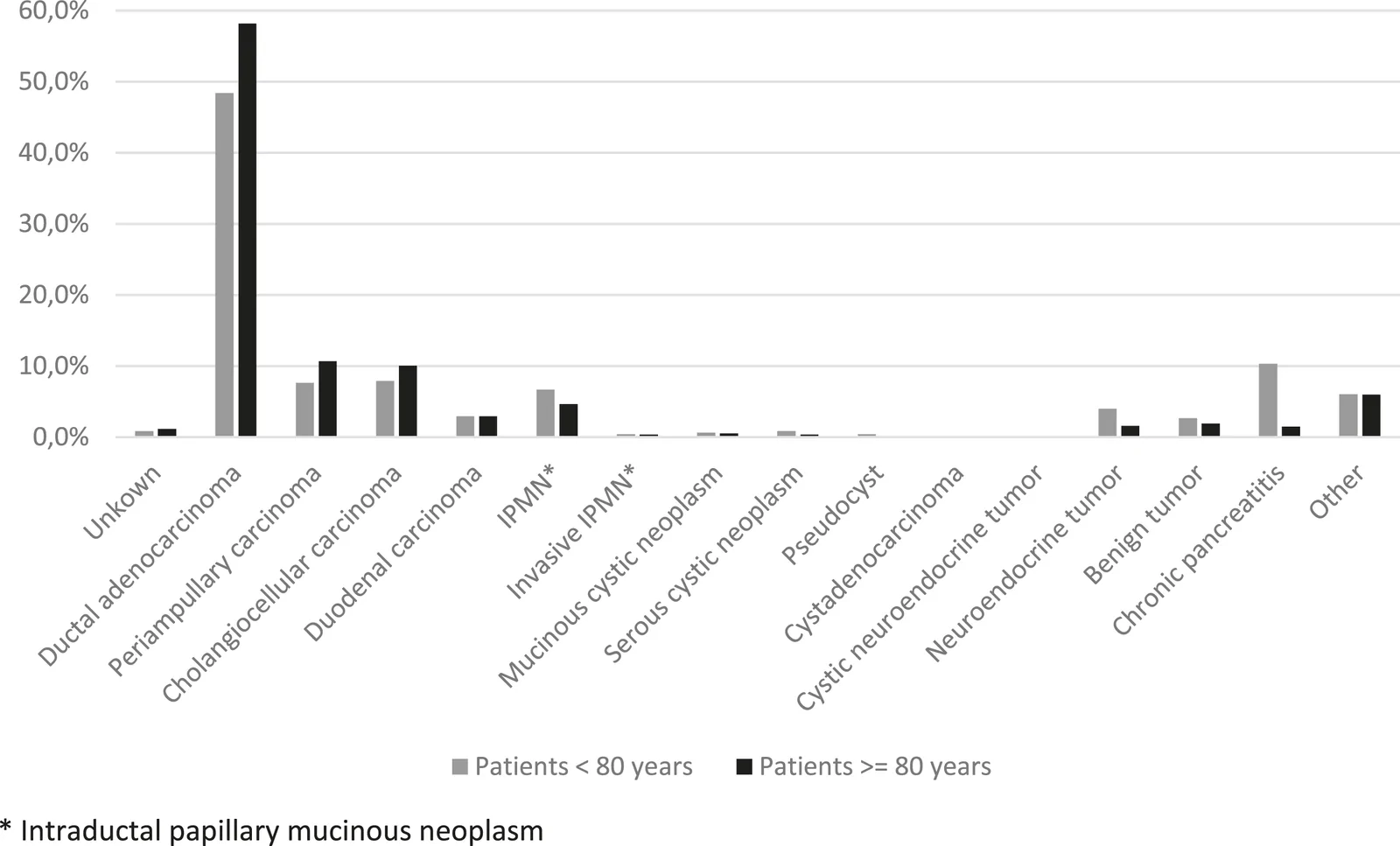
Histopathological analysis of the specimens.

Both in-hospital and 30-day mortality were significantly higher in patients ≥80 years than in younger patients: 8.2% vs. 4.4% and 7.2% vs. 3.6%, respectively. While surgical complications were the most common cause of death within 30 days of surgery in patients <80 years of age, nonsurgical complications predominated in very elderly patients (Table 3).

**Table 3.** Postoperative parameters

​	Patients < 80 years, *n* (%)	Patients ≥ 80 years, *n* (%)	*p* value
Postoperative complication(s)	6,520 (60.7)	1,002 (64.0)	0.013
Severe postoperative complication(s)	2,906 (28.3)	403 (28.0)	0.836
Pancreatic fistula	2,388 (22.2)	230 (20.4)	0.359
Biochemical leak	778 (7.2)	97 (6.2)	​
Grade B	1,012 (9.4)	141 (9.0)	​
Grade C	598 (5.6)	82 (5.2)	​
Delayed gastric emptying	2,190 (20.4)	372 (23.8)	**0.017**
Postoperative hemorrhage	1,240 (11.5)	196 (12.5)	0.555
Bile fistula	583 (5.4)	75 (4.8)	0.293
Wound dehiscence	214 (2.0)	38 (2.4)	0.257
Postoperative ventilation > 48 h	642 (6.0)	112 (7.2)	0.070
Pneumonia	598 (5.6)	129 (8.2)	**<0.001**
Pulmonary embolism	216 (2.0)	25 (1.6)	0.268
Stroke	51 (0.5)	9 (0.6)	0.596
Myocardial infarction	68 (0.6)	17 (1.1)	**0.043**
Postoperative ICU stay (med [min/max])	2 (0/99)	3 (0/77)	<0.001
Postoperative hospital stay (med [min/max])	16 (0/917)	18 (1/150)	<0.001
In-hospital mortality	473 (4.4)	129 (8.2)	<0.001
30-day mortality	384 (3.6)	112 (7.2)	<0.001
Primary disease	17 (0.2)	10 (0.6)	​
Surgical complication	173 (1.6)	42 (2.7)	​
Non-surgical complication	131 (1.2)	44 (2.9)	​
Other reasons/unknown	63 (0.6)	16 (1.0)	​

Postoperative complications occurred more frequently in very elderly patients (64% vs. 60.7%, *p* = 0.013). In contrast, there was no difference between the two groups in terms of severe postoperative complications (Clavien-Dindo grade 3 and 4) (Table 3). Postoperative pancreatic fistula developed in approximately one in five patients, with no difference between the groups. Similarly, there were no statistically significant differences between the two groups in other surgical complications such as biliary fistulas, wound healing impairments, or postoperative hemorrhages. In contrast, cardiopulmonary complications such as pneumonia (8.2% vs. 5.6%, *p* < 0.001) or myocardial infarction (1.1% vs. 0.6%, *p* < 0.001) occurred significantly more frequently in the very elderly group. After surgery, very elderly patients spent an average of one additional day in intensive care and two additional days in the hospital (Table 3).

In a further step, the aforementioned demographic and intraoperative parameters were analyzed for their association with in-hospital mortality (Table 4). In the multivariate analysis, age ≥ 80 years, male sex, overweight (body mass index >25 kg/m^2^), ASA class 3 or 4, the coincidence of COPD or conditions requiring anticoagulant therapy, the need for intraoperative blood transfusions, and extended resections were identified as independent risk factors for in-hospital mortality (Table 4). In a propensity score-matched cohort comprising 1,566 matched pairs with optimal covariate balance, age ≥ 80 years was not independently associated with in-hospital mortality. Conditional logistic regression yielded an odds ratio of 1.10 (95% CI: 0.85–1.44, *p* = 0.461). These findings were confirmed by a paired McNemar’s test (*p* = 0.50).

**Table 4.** Uni-, multivariate analysis for in-hospital mortality

​	Univariate analysis		OR	Multivariate analysis	
	mortality in-hospital, *n* (%)	*p* value		95% CI	*p* value
Age					
<80 years	473 (4.4)	**<0.001**	0.547	0.439–0.683	**<0.001**
≥80 years	129 (8.2)	​	​	​	​
Gender					
F	196 (3.6)	**<0.001**	0.621	0.515–0.750	**<0.001**
M	406 (5.9)	​	​	​	​
BMI					
≤25 kg/m^2^	260 (4.1)	**<0.001**	0.742	0.622–0.885	**<0.001**
>25 kg/m	342 (5.8)	​	​	​	​
ASA					
3/4	412 (6.4)	**<0.001**	1.664	1.365–2.028	**<0.001**
1/2	188 (3.2)	​	​	​	​
Diabetes mellitus					
Yes	169 (5.4)	0.152	​	​	​
No	433 (4.7)	​	​	​	​
Coronary heart disease					
Yes	130 (7.9)	**<0.001**	1.210	0.954–1.535	0.116
No	474/4.4	​	​	​	​
Heart failure					
Yes	106 (7.6)	**<0.001**	1.121	0.897–1.401	0.315
No	476 (4.4)	​	​	​	​
COPD					
Yes	56 (9.1)	**<0.001**	1.827	1.352–2.469	**<0.001**
No	546 (4.7)	​	​	​	​
Anticoagulant therapy					
Yes	172 (7.2)	**<0.001**	1.281	1.037–1.582	**0.022**
No	430 (4.3)	​	​	​	​
Surgical procedure					
pp-Whipple	409 (4.6)	**0.019**	1.036	0.854–1.257	0.717
Classical Whipple	193 (5.6)	​	​	​	​
Pancreatic anastomosis					
PG	118 (5.6)	**0.057**	1.001	0.991–1.011	0.824
PJ	471 (4.7)	​	​	​	​
Extended resection					
Yes	94 (9.4)	**<0.001**	2.044	1.658–2.755	**<0.001**
No	508 (4.7)	​	​	​	​
Resection portal vein					
Yes	73 (5.5)	0.295	​	​	​
No	529 (4.8)	​	​	​	​
Resection truncus coeliacus^a^					
Yes	2 (5)	0.975	​	​	​
No	600 (4.9)	​	​	​	​
Intraoperative blood transfusion					
Yes	124 (10.7)	**<0.001**	2.395	1.913–2.998	**<0.001**
No	478 (4.3)	​	​	​	​
Malignant disease					
Yes	445 (4.9)	**0.046**	0.994	0.975–1.010	0.413
No	98 (4.0)	​	​	​	​

^a^ Resection of the truncus coeliacus refers to highly selected extended pancreatoduodenectomies in which resection of the truncus coeliacus was performed in conjunction with pancreatoduodenectomy, followed by reconstruction of the hepatic arterial inflow (e.g., hepatic artery reconstruction).

To identify risk factors specifically associated with mortality in very elderly patients, an additional subgroup analysis was performed including only patients aged ≥ 80 years. In univariate analysis, ASA class 3 or 4 (9.2% vs. 5.8%, *p* = 0.025), the presence of coronary heart disease (10.9% vs. 7.4%, *p* = 0.034), male sex (11.5% vs. 5.1%, *p* < 0.001), and the need for postoperative blood transfusions (18.5% vs. 6.9%, *p* < 0.001) were significantly associated with in-hospital mortality in this subgroup. In the subsequent multivariate analysis restricted to patients aged ≥ 80 years, male sex (odds ratio: 2.433, 95% CI: 1.592–3.717, *p* < 0.001) and the intraoperative blood transfusion rate (odds ratio: 3.195, 95% CI: 2.013–5.071, *p* < 0.001) remained independent risk factors for increased in-hospital mortality, whereas ASA class 3 or 4 and coronary heart disease did not retain statistical significance.

## Discussion

All pancreatoduodenectomies recorded in the DGAV|registry pancreas up to April 2024 were included in this retrospective study. The analysis of these 12,305 surgeries therefore provides a reliable reflection of the quality of care and treatment in centers for pancreatic surgery in Germany.

During the more than 12.5-year study period, the number of pancreatoduodenectomies registered annually in the DGAV|registry pancreas more than doubled, reaching a peak value of 1,587 procedures in 2021. This increase is partly attributed to the growing number of pancreatic centers reporting their data to the DGAV|registry pancreas. Furthermore, it reflects the trend toward centralization with specialized centers having concentrated surgical expertise as well as specific experience and equipment for perioperative care (clinical pathways, ERAS, and prehabilitation programs) [6, 11]. Moreover, the proportion of very elderly patients undergoing surgery during the same period more than doubled, rising from approximately 7% in 2014 to 15.9% at the end of the study period. This trend reflects the demographic development in visceral surgery, but particularly in pancreatic surgery, toward an increasingly older patient population [12].

The decision to use an age cutoff of 80 years for the present analysis reflects a deliberate focus on very elderly patients rather than on elderly patients in general. While some previous studies have applied lower thresholds such as 75 years, these often encompass a heterogeneous population in which chronological age alone is less strongly correlated with frailty and physiological reserve. In contrast, age ≥ 80 years more consistently reflects advanced biological vulnerability, which is evident in our cohort by the markedly higher prevalence of severe comorbidities and higher ASA classifications [13].

Furthermore, notable differences between the groups emerged regarding surgical indication and histopathological findings. Malignant diseases, particularly pancreatic ductal adenocarcinoma, occurred significantly more often in patients aged 80 years and older. This may reflect, on the one hand, epidemiological trends, indicating an increasing age-standardized incidence of pancreatic ductal adenocarcinoma in older age groups [7]. On the other hand, this observation suggests a selective indication with the procedure being considered primarily as a potentially curative treatment for malignant diseases in the very elderly patients, while more conservative approaches are pursued for benign diseases in this patient group [1].

The analysis of the data revealed a significantly higher in-hospital (8.2% vs. 4.4%) and 30-day mortality (7.2% vs. 3.6%) in the very elderly patients. A large systematic review and meta-analysis of over 22,481 patients undergoing pancreatoduodenectomy reported similar findings, with octogenarians having a twofold higher risk of mortality [6]. These results suggest that pancreatectomy in very elderly patients, as seen in our cohort, is predominantly performed for (potentially) malignant indications for which there are currently no curative nonsurgical alternatives [14].

Various publications highlighted the association between increased peri- and postoperative mortality in very elderly patients and the higher incidence and severity of concomitant diseases [1, 12, 15]. In our cohort, cardiovascular comorbidities and diabetes mellitus occurred significantly more frequently in the very elderly patients. Furthermore, more than two thirds of these patients were categorized as ASA classes 3 or 4 (vs. 50% of the younger patients, *p* < 0.001). This emphasizes the clinical relevance and severity of these comorbidities.

In the multivariate analysis, in addition to age ≥ 80 years, male sex, obesity, ASA class 3 or 4, COPD, anticoagulant therapy, the need for intraoperative blood transfusions, and extended pancreatic resections were identified as independent risk factors. These results underscore the correlation between patients’ overall health status shaped by pre-existing and concomitant diseases and mortality [15].

To further address the heterogeneity inherent in this registry-based cohort, we performed a propensity score-matched sensitivity analysis comparing patients aged ≥ 80 years with younger patients of comparable preoperative risk profiles. In contrast to the multivariate analysis of the overall cohort, chronological age ≥ 80 years was no longer independently associated with in-hospital mortality after matching.

The absence of a significant association between advanced age and in-hospital mortality in this analysis underscores the concept that biological rather than chronological age may be the key determinant of early postoperative outcomes following pancreatic head resection [13]. These findings support a risk-adapted, individualized surgical decision-making process and caution against the use of chronological age alone as a contraindication for major pancreatic surgery [16, 17].

When the analysis was restricted to the subgroup of patients aged ≥ 80 years, most factors identified in the overall cohort – including ASA class and coronary heart disease – were no longer independently associated with mortality in multivariable analysis. In contrast, within this population, which is inherently characterized by a higher prevalence and severity of comorbid conditions, male sex and the need for intraoperative blood transfusions emerged as independent risk factors in this very elderly cohort. The association between male sex and adverse outcomes is consistent with previous studies by Merath et al. [2] and Roalsø et al. [18], which reported increased morbidity and mortality following pancreatic surgery in male patients. Hormonal and subsequent immunological differences between the sexes have been proposed as potential underlying mechanisms [19]. The independent impact of intraoperative blood transfusions may reflect increased surgical complexity, perioperative blood loss, or transfusion-related immunomodulatory effects, all of which may disproportionately affect very elderly patients [20].

The incidence of postoperative complications was significantly higher in patients ≥ 80 years (64.0% vs. 60.7%, *p* = 0.013). These findings are consistent with previous studies and meta-analysis that indicate increased morbidity in very elderly [6, 21]. However, the present study found no significant difference between the two groups in terms of severe or surgically relevant complications of Clavien-Dindo grades 3 and 4 (28% vs. 28.3%). Likewise, the rate of specific surgical complications, including postoperative pancreatic fistula, bile fistula, postoperative hemorrhage, and wound healing impairments, did not differ significantly. Similarly, a meta-analysis by Phillipos et al. [6] found no difference in surgically relevant complications after pancreatoduodenectomy between patients ≥80 years and younger individuals. There is evidence that an increased firmness of pancreatic tissue and uniform dilation of the pancreatic duct with increasing age may reduce the risk of pancreatic fistulas [22]. In contrast, delayed gastric emptying occurred significantly more frequently in the very elderly cohort [17]. Given comparable rates of fistulas and local septic complications, this may be attributable to a generally reduced gastrointestinal motility in older patients [23].

In contrast to surgical complications, postoperative pneumonia (8.2% vs. 5.6%) and myocardial infarction (1.1% vs. 0.6%) occurred significantly more frequently in patients aged 80 years and older. Phillipos et al. [6] likewise reported a twofold increase in postoperative cardiac or respiratory complications in patients ≥80 years. These findings are consistent with other studies that attribute the increased postoperative risk to pre-existing pulmonary and cardiac disease [17, 21].

Both postoperative intensive care unit stay and overall hospital stay were significantly longer in patients ≥80 years, likely reflecting a more complex postoperative course and higher incidence of postoperative complications in these patients**.** Previous studies have reported similar findings, with Langan et al. [24] and Dale et al. [25] additionally highlighting the increased hospital costs and greater rehabilitation needs of elderly and very elderly patients.

This study has several limitations. Patients in the DGAV|registry pancreas cannot be assigned to individual centers due to anonymized data entry. As a result, center-specific case volumes are not available, and stratification into low- and high-volume centers or analysis of volume-outcome relationships is not possible. This introduces heterogeneity not only in the surgical volume and surgeon experience**,** both known to strongly influence postoperative outcomes [26], but also in patient selection and peri- and postoperative management strategies (e.g., ERAS programs, clinical pathways, prehabilitation programs). Furthermore, substantial variability exists between centers, for example, with regard to the different reconstruction techniques (pancreatogastrostomy vs. pancreatojejunostomy) and their modifications, as well as the type of surgical approach, open or minimally invasive. The registry does not provide information on specific inclusion and exclusion criteria for open versus minimally invasive procedures and also does not document the rationale for choosing a particular reconstruction technique. However, for the purpose of our study, comparing postoperative morbidity and mortality between patients aged <80 vs. ≥80 years and describing the current practice in Germany, these factors are of secondary relevance.

Another point concerns the reported age cutoff of 80 years itself. While a more granular age stratification (e.g., distinguishing between elderly and very elderly patients) might provide additional insights, the present study was specifically designed to evaluate outcomes in patients aged ≥80 years as a clinically distinct subgroup. Future studies incorporating standardized measures of functional status and frailty may allow for more refined risk stratification beyond chronological age alone.

Finally, data on patients’ ECOG performance status were not consistently recorded. While the ASA score captures comorbidities and perioperative risk, it does not fully reflect functional status or frailty, which are known to influence postoperative outcomes and overall survival, especially in elderly patients [27]. Inclusion of standardized performance status measures such as ECOG in future registry updates would allow more comprehensive risk stratification and individualized surgical decision-making. Despite these limitations, the large multicenter cohort provides a robust and representative overview of contemporary pancreatic surgery in Germany, supporting the conclusion that age alone should not be considered an exclusion criterion for pancreatoduodenectomy.

### Summary

The growing number of pancreatoduodenectomies in patients ≥ 80 years demonstrates that chronological age alone is not, and should not be, an exclusion criterion for surgery. Careful patient selection that considers comorbidities, physical condition, and patient preferences is essential. Incorporating geriatric expertise and strategies to improve patients’ capability to surgery, such as prehabilitation programs, as integral components of a multimodal treatment approach may represent promising strategies to improve peri- and postoperative outcomes in very elderly patients undergoing pancreatoduodenectomy.

## Acknowledgments

This work has been conducted using the DGAV|registry pancreas provided by the German society for general and visceral surgery (Deutsche Gesellschaft für Allgemein- und Viszeralchirurgie [DGAV]).

## Statement of Ethics

Patient consent for the present study was not required, as this was a retrospective analysis of data for which the patients had already provided consent for collection at the participating study centers. The study was submitted to the Ethics Committee of the Medical Association of Saarland (Ärztekammer des Saarlandes) and received approval (reference number: 158/25, date: 10/09/2025). Written informed consent from participants was not required in accordance with local/national guidelines.

## Conflict of Interest Statement

Prof. Tobias Keck was a member of the journal’s Editorial Board, and Prof. Jens Werner was one of the editors-in-chief at the time of submission. The other authors have no conflicts of interest to declare.

## Funding Sources

This study was not supported by any sponsor or funder.

## Author Contributions

P.S.: formal analysis, investigation, methodology, visualization, and writing – original draft. G.G., W.U., H.B., J.C.K., J.W., T.K., J.-P.R., D.I., A.P., S.F.-F., C.R., M.Gh., and S.H.: writing – review and editing. P.J.: formal analysis, software, and writing – review and editing. C.K.: data curation and writing – review and editing. M.Gl.: conceptualization, project administration, supervision, validation, and writing – review and editing.

## Data Availability

The data that support the findings of this study are not publicly available due their containing information that could compromise the privacy of research participants but are available from the DGAV|registry pancreas of the German Society for General and Visceral Surgery (DGAV). Further inquiries can be directed to the corresponding author.
